# Dietary ginger as a traditional therapy for blood sugar control in patients with type 2 diabetes mellitus: A systematic review and meta-analysis

**DOI:** 10.1097/MD.0000000000015054

**Published:** 2019-03-15

**Authors:** Fang-yan Huang, Ting Deng, Lian-xin Meng, Xin-ling Ma

**Affiliations:** Youjiang Medical University for Nationalities, Baise, Guangxi, PR China.

**Keywords:** fasting blood sugar, ginger consumption, glycated hemoglobin, natural therapy, type 2 diabetes mellitus

## Abstract

**Background:**

Ancient medical practitioners used to encourage dietary supplements and herbal medicine for the treatment of type 2 diabetes mellitus (T2DM). Ginger (*Zingiber officinale*), is a nontoxic spice with negligible side effects, and is considered safe by the food and drug administration. In this analysis, we aimed to systematically compare fasting blood sugar (FBS) and glycated hemoglobin (HbA1c) at baseline versus at follow-up in T2DM patients who consumed and who did not consume ginger.

**Methods:**

A literature search was carried out through MEDLINE, Embase, the Cochrane Central, and www.ClinicalTrials.gov for English-published trials comparing glucose parameters in T2DM patients who were assigned to ginger consumption versus a control group. All the participants were patients with T2DM who were either assigned to ginger therapy (1600– 4000 mg daily) or to a control group. FBS and HbA1c were assessed in the ginger and control groups, respectively, from baseline to follow-up to observe any significant change. Weight mean difference (WMD) with 95% confidence intervals (CI) was calculated to represent the analysis which was carried out by the RevMan 5.3 software.

**Results:**

Eight randomized trials consisting of a total number of 454 participants with T2DM were included in this analysis. At first, FBS was compared in patients with T2DM from baseline prior to ginger consumption until follow-up after ginger consumption. The results showed no significant difference in FBS (WMD: 1.38, 95% CI: [−0.53–3.30]; *P = *.16). For the T2DM patients who did not consume ginger, no significant difference in FBS was observed (WMD: −0.27, 95% CI: [−5.09–4.54]; *P = *.91). However, a significantly improved HbA1c from baseline to follow-up was observed in those participants with ginger consumption (WMD: 0.46, 95% CI: [0.09–0.84]; *P = *.02) whereas in the control group, no significant difference in HbA1c was observed (WMD: −0.23, 95% CI: [−0.60–0.14]; *P = *.22).

**Conclusion:**

This analysis involving patients with T2DM showed no significant difference in FBS with ginger consumption. However, dietary ginger significantly improved HbA1c from baseline to follow-up showing that this natural medicine might have an impact on glucose control over a longer period of time in patients with T2DM.

## 1 Introduction

Type 2 diabetes mellitus (T2DM) is still a global issue in this era of 2018. Several new therapies have been tried to maintain the blood sugar level to a normal level^[1–4]^ in order to prevent complications associated with this chronic disease. However, oral antidiabetic medications might sometimes be associated with unwanted side effects leading to drug discontinuation.^[5]^ In order to improve this situation, diabetes therapies associated with less adverse events would be required.^[6]^

From the perception of Chinese medicine, an individual categorized as having an unhealthy body constitution would develop progressive chronic disorders such as diabetes mellitus and heart diseases. Yin-deficiency, Yang-deficiency, and Yin-Yang deficiencies were observed in patients with T2DM. Hence, ancient medical practitioners and elderly Chinese people used to encourage dietary supplements and herbal medicine for the treatment of T2DM.^[7]^ Traditional Chinese medicine has shown vital benefits in the treatment of patients with T2DM and other diseases.^[8–13]^ Spices have long been known for their anti-inflammatory, antioxidant, and antidiabetic properties.^[14]^ Today, new researchers are interested to further discover and explore the biopharmaceutical activities of these dietary supplements.^[15–16]^

Ginger, also known as *Zingiber officinale*, is a nontoxic spice with negligible side effects, and is considered safe by the food and drug administration (FDA).^[17]^ Several researches have shown ginger to be beneficial in patients with T2DM.^[18]^ However, most of the studies were literature and systematic reviews lacking data evidence. There was a need for data analysis, which, with evidence, could probably show the beneficial effects of ginger in patients with T2DM.

In this analysis, we aimed to systematically compare fasting blood sugar (FBS) and glycated hemoglobin (HbA1c) at baseline versus at follow-up in T2DM patients who consumed and who did not consume ginger.

## 2 Methods

### 2.1 Literature search: searched databases, searched terms, inclusion and exclusion criteria

A literature search was carried out through MEDLINE (PubMed), Embase, the Cochrane Central database, and www.ClinicalTrials.gov for English-published trials (until July 2018) comparing glucose parameters in T2DM patients who were assigned to ginger supplement and a control group respectively.

The following search terms were used:

- Ginger and type 2 diabetes mellitus;
- Ginger and glucose control;
- Ginger and T2DM;
- Ginger and diabetes mellitus;
- *Zingiber officinale* and diabetes mellitus;
- *Zingiber officinale* and glucose control;
- *Zingiber officinale* and type 2 diabetes mellitus.

The following inclusion criteria were considered:

- Randomized trials involving patients with T2DM;
- Trials comparing FBS and HbA1c in participants who were assigned to a ginger and a control group;
- Trials reporting FBS and HbA1c at baseline and at follow-up.

The following exclusion criteria were considered:

- Nonrandomized trials, systematic reviews, meta-analyses, and case studies;
- Trials which did not involve patients with T2DM;
- Trials that did not report FBS and HbA1c;
- Trials that were not based on patients who were assigned to ginger supplements;
- Trials that included data which could not be used in this meta-analysis;
- Duplicated studies.

### 2.2 Participants, endpoint, and follow-up time period

All the participants were patients with T2DM who were either assigned to ginger therapy (1600– 4000 mg daily) or to a control group as shown in Table 1.

**Table 1:**
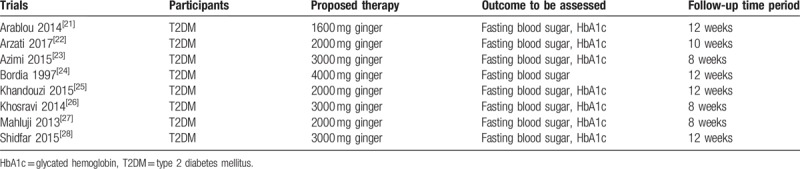
Outcomes, follow-up time periods.

Fasting blood sugar and HbA1c were assessed in the ginger and the control groups, respectively, at baseline versus at follow-up to observe for any significant change.

The significance of fasting blood sugar was to check whether blood glucose was under control on a daily basis.

The significance of HbA1c was to check blood sugar control over a longer period of time (1–2 months).

A follow-up time period of 8 to 12 weeks were considered relevant to this meta-analysis as shown in Table 1.

### 2.3 Data extraction and quality assessment

The type of study, the total number of T2DM participants assigned to the ginger and the control groups, the respective average FBS and HbA1c which were reported, and the baseline features of the participants were carefully extracted by 4 authors (FYH, TD, LXM, and XLM). Any disagreement which followed was considerately discussed and then solved by the corresponding author.

The quality assessment of the trials was carried out with reference to the criteria suggested by the Cochrane Collaboration.^[19]^

Sensitivity analysis was carried out by a method of exclusion.

### 2.4 Statistical analysis

Data that were extracted to be used in this analysis consisted of mean, standard deviation (sd), and the number of participants from each trial. For the continuous variable, weight mean difference (WMD) with 95% confidence intervals (CI) was calculated to represent the analysis which was carried out by the RevMan 5.3 software.

Heterogeneity was assessed by the *Q* statistic test whereby a result was considered statistically significant if the *P* value obtained was less or equal to .05.

Heterogeneity was also assessed by the *I*^2^ test, whereby a higher *I*^2^ represented a higher heterogeneity.

The statistic effect models which were used during the analysis included a fixed effect model (*I*^2^ < 50%) or a random effect model (*I*^2^ > 50%).

### 2.5 Compliance with ethical guidelines

Ethical approval was not required for this study since it did not involve experiments with animals or humans performed by any of the authors.

## 3 Results

### 3.1 Search outcomes

The PRISMA reporting guideline was applied.^[20]^ Search databases resulted in a total number of 205 publications. Following an initial assessment, 178 publications were eliminated due to nonrelevance.

Twenty-seven (27) full text articles were assessed for eligibility.

Further assessment of the full-text articles was carried out and more publications were eliminated for the following reasons:

- They were systematic reviews and meta-analyses (2);
- They were observational cohorts and case studies (4);
- They were based on healthy volunteers (1);
- They were duplicated studies (12).

**Figure 1:**
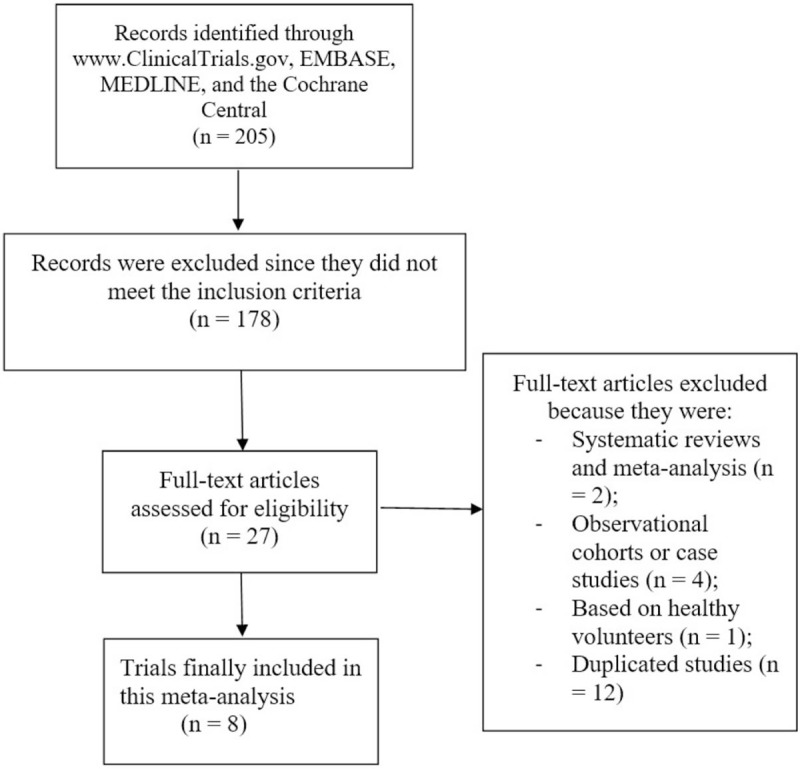
Flow diagram representing the flow of study.

Finally, 8 trials^[21–28]^ were included in this analysis as shown in Figure 1.

### 3.2 Main features of the trials and baseline characteristics of the participants

Eight randomized trials with a total number of 454 participants with T2DM were included in this analysis. Two hundred and forty five (245) participants were assigned to the ginger group whereas 209 participants were assigned to the control group. The data were represented in Table 2.

**Table 2:**
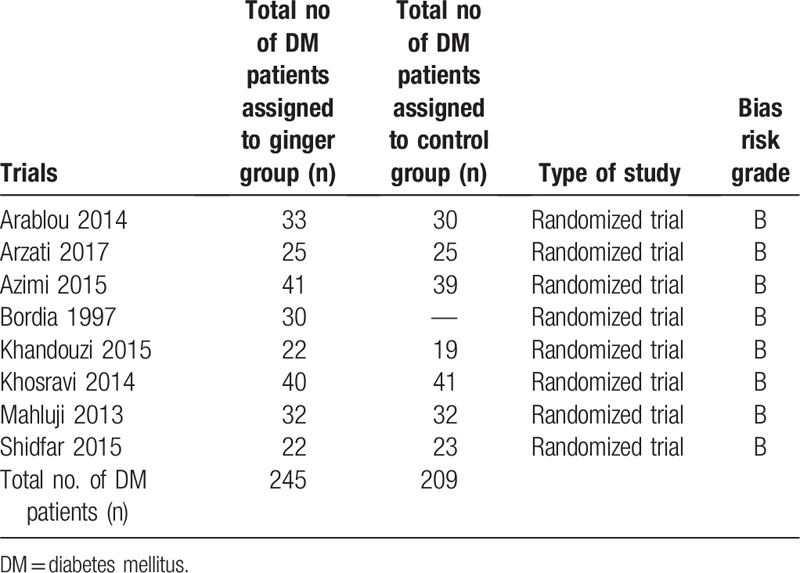
General features of the studies.

In addition, following the methodological assessment, a grade B was allotted to all the trials based on the criteria suggested by the Cochrane Collaboration.

The baseline features of the participants were reported in Table 3. At baseline, patients had an HbA1c ranging from 6.90% to 8.40%. The participants had a mean age ranging from 45.2 to 55.2 years. The percentage of male T2DM patients as well as the average body mass index (BMI) of the participants have been listed in Table 3.

**Table 3:**
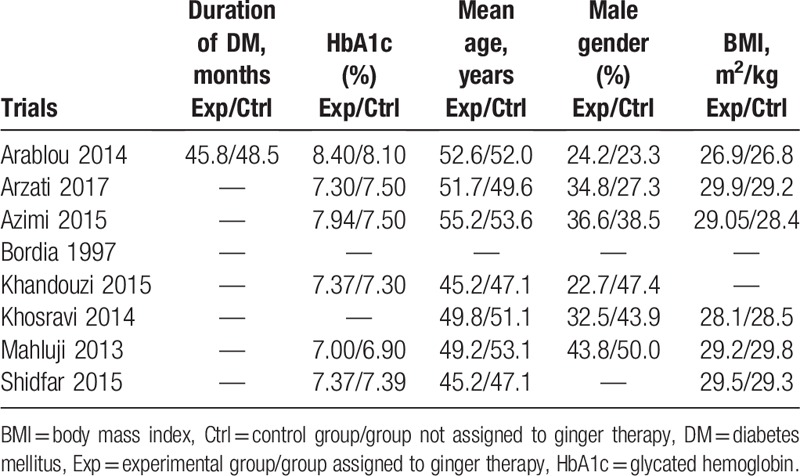
Baseline features of the participants.

### 3.3 Fasting blood glucose in patients with type 2 diabetes mellitus assigned to the ginger consumption group

**Figure 2:**
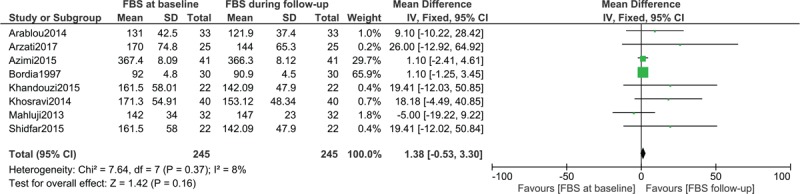
Fasting blood glucose in patients with type 2 diabetes mellitus assigned to the Ginger consumption group.

First of all, FBS was compared in patients with T2DM from baseline prior to ginger consumption to follow-up after ginger consumption. The analysis included a total number of 245 participants with T2DM. The results showed no significant difference in FBS with WMD: 1.38, 95% CI: [−0.53–3.30]; *P = *.16 as shown in Figure 2.

### 3.4 Fasting blood glucose in patients with type 2 diabetes mellitus assigned to the Control group

**Figure 3:**
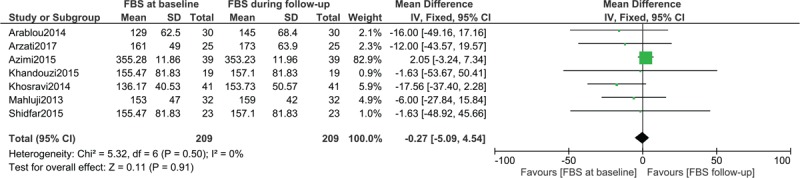
Fasting blood glucose in patients with type 2 diabetes mellitus assigned to the control group.

FBS was also assessed in 209 patients with T2DM who did not consume ginger supplement. The result showed no significant difference in FBS at baseline and at follow-up with WMD: −0.27, 95% CI: [−5.09–4.54]; *P = *.91 as shown in Figure 3.

### 3.5 HbA1c in patients with type 2 diabetes mellitus assigned to the ginger consumption group

**Figure 4:**
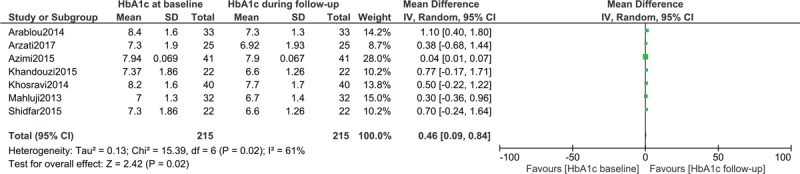
HbA1c in patients with type 2 diabetes mellitus assigned to the Ginger consumption group. HbA1c = glycated haemoglobin.

HbA1c from baseline to follow-up was also assessed in patients with T2DM who were assigned to the ginger consumption group. Two hundred and fifteen participants with T2DM were assessed. The results showed a significantly improved HbA1c from baseline to follow-up with WMD: 0.46, 95% CI: [0.09–0.84]; *P = *.02 as shown in Figure 4.

### 3.6 HbA1c in patients with type 2 diabetes mellitus assigned to the Control group

**Figure 5:**
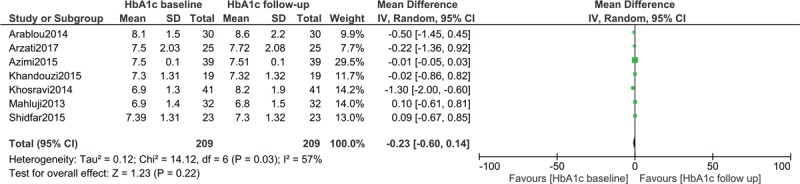
HbA1c in patients with type 2 diabetes mellitus assigned to the control group. HbA1c = glycated haemoglobin.

HbA1c from baseline to follow-up was also assessed in 209 patients with T2DM who were assigned to the control group. Results showed no significant difference in HbA1c within the control group with WMD: −0.23, 95% CI: [−0.60–0.14]; *P = *.22 as shown in Figure 5.

## 4 Discussion

The results of this meta-analysis showed with evidence, the benefits of ginger intake to control blood sugar level in patients with T2DM, especially during the long term. Ginger consumption was not associated with increased FBS. However, HbA1c significantly improved from baseline to follow-up in these patients with T2DM.

Another systematic review and meta-analysis showed beneficial effects of ginger consumption in patients with T2DM.^[29]^ The analysis showed that ginger subsequently reduced FBS and significantly improved HbA1c in these patients with T2DM which was partly in support of this current analysis. However, their analysis was not strictly based on diabetic control and our analysis was better in the way that it included even more trials to assess the corresponding endpoints as compared to the previous meta-analysis.

Another study showed that consumption of ginger (1000 mg daily) might reduce plasma fasting sugar thus preventing complications such as hyper insulinemia, dyslipidemia, peritoneal membrane fibrosis, and cardiovascular disease in patients on peritoneal dialysis.^[30]^ Ginger also has beneficial effects on obesity and metabolic syndrome.

Ginger has antidiabetic properties and studies have shown ginger to control hyperinsulinemia in patients with T2DM.^[31]^ Ginger also has potential effects in preventing or reducing diabetic complications such as micro-vascular retinopathy.^[32]^ Additionally, ginger has shown to protect the liver, kidney, and neural system complications in patients with T2DM. The mechanisms which are involved deal with insulin release and increased plus accelerated carbohydrate and lipid metabolism.^[33]^

Furthermore, ginger consumption was associated with other potential benefits especially in patients with chronic diseases.^[34]^ Apart from T2DM, ginger was shown to be effective in patients with hypertension and coronary artery disease.^[35]^ This natural traditional medicine could even act as a primary preventive measure to these chronic disorders.

### 4.1 Limitations

The restricted total number of participants could be a major limitation of this study. Secondly, follow-up time period varied from 8 to 12 weeks, even if to a lesser extent, this could have influenced the results. In addition, the duration of disease was unknown in most of the studies. It is also not known whether the participants were previously or recently diagnosed with T2DM. The daily amount of ginger which were different in different trials might also be a limiting factor in this study. There was also no major mention about the consumption of western medicine which might have influenced the final outcomes.

## 5 Conclusion

This analysis involving patients with T2DM showed no significant difference in FBS with ginger consumption. However, dietary ginger significantly improved HbA1c from baseline to follow-up showing that this natural medicine might have an impact on glucose control over a longer period of time in patients with T2DM.

## Acknowledgment

All named authors meet the International Committee of Medical Journal Editors (ICMJE) criteria for authorship for this article, take responsibility for the integrity of the work as a whole, and have given their approval for this version to be published.

## Author contributions

Fang-yan Huang, Ting Deng, Lian-xin Meng, and Xin-ling Ma were responsible for the conception and design, acquisition of data, analysis and interpretation of data, drafting the initial manuscript and revising it critically for important intellectual content. Fang-yan Huang wrote this manuscript.

**Conceptualization:** Fang-yan Huang, Ting Deng, Lian-xin Meng, Xin-ling Ma.

**Data curation:** Fang-yan Huang, Ting Deng, Lian-xin Meng, Xin-ling Ma.

**Formal analysis:** Fang-yan Huang, Ting Deng, Lian-xin Meng, Xin-ling Ma.

**Funding acquisition:** Fang-yan Huang, Ting Deng, Lian-xin Meng, Xin-ling Ma.

**Investigation:** Fang-yan Huang, Ting Deng, Lian-xin Meng, Xin-ling Ma.

**Methodology:** Fang-yan Huang, Ting Deng, Lian-xin Meng, Xin-ling Ma.

**Project administration:** Fang-yan Huang, Ting Deng, Lian-xin Meng, Xin-ling Ma.

**Resources:** Fang-yan Huang, Ting Deng, Lian-xin Meng, Xin-ling Ma.

**Software:** Fang-yan Huang, Ting Deng, Lian-xin Meng, Xin-ling Ma.

**Supervision:** Fang-yan Huang, Ting Deng, Lian-xin Meng, Xin-ling Ma.

**Validation:** Fang-yan Huang, Ting Deng, Lian-xin Meng, Xin-ling Ma.

**Visualization:** Fang-yan Huang, Ting Deng, Lian-xin Meng, Xin-ling Ma.

**Writing – Original Draft:** Fang-yan Huang, Ting Deng, Lian-xin Meng, Xin-ling Ma.

**Writing – Review & Editing:** Fang-yan Huang, Ting Deng, Lian-xin Meng, Xin-ling Ma.
